# Effect of incorporation of tricalcium silicate to a universal adhesive on microtensile bond strength to dentin and micromorphological patterns of tooth/restoration interface

**DOI:** 10.1038/s41598-026-44781-1

**Published:** 2026-04-09

**Authors:** Tarek Zayed, Naglaa Elkholany, Amira Motawea, Hamdi Hamama

**Affiliations:** 1 Department of Conservative Dentistry, Faculty of Dentistry, Mansoura University, Mansoura, 35516 Egypt; 2 Department of Pharmaceutics, Faculty of Pharmacy, Mansoura University, 35516 Mansoura, Egypt; 3 Department of Pharmaceutics, Faculty of Pharmacy, Mansoura National University, 7731168 Gamasa, Egypt; 4 Faculty of Oral and Dental Medicine, Alsalam University, 6618002 Tanta, Egypt

**Keywords:** Universal adhesives, Dentin, Bond strength, Bioactive, Tri calcium silicate, Microtensile bond strength, Micromorphology, Health care, Materials science, Medical research

## Abstract

The durability of the resin-dentin interface in the oral environment is an important concern in bonded restorations. This study aimed to evaluate and compare the effect of incorporation of tricalcium silicate (TCS) to a universal adhesive on microtensile bond strength (µTBS) to dentin. Moreover, this study was designed to evaluate micromorphological patterns of tooth/restoration interface, immediately after 24 h and after 6 months storage. Forty permanent human molars were selected and randomly assigned into two equal groups (*n* = 20) according to the incorporation of TCS to the universal adhesive; Group1: Control group without incorporation of TCS. Group2: with the incorporation of TCS to the universal adhesive. Each group was further subdivided into immediate and delayed testing subgroups according to the testing time. The adhesives were applied according to the manufacturers’ instructions. Composite/dentin beams were prepared (1 mm^2^). The samples were subjected to µTBS testing. Three restored specimens from each group were utilized in micromorphological evaluation of resin-dentin interfaces using Scanning Electron Microscope (SEM). µTBS data were statistically analyzed using two-way ANOVA and multiple comparison post hoc tests. The outcome of Two-way ANOVA for the µTBS test revealed that both the incorporation of TCS and time had statistically significant differences in the µTBS (*p* < 0.05). There was no significant difference in µTBS between the control and TCS groups immediately, according to the Tukey post hoc multiple comparison test (*p* > 0.05). Nonetheless, µTBS significantly declined in the delayed control group. Additionally, all tested groups had a statistically significant difference when compared to the delayed control adhesive group (*p* < 0.05). The hybrid layers made with both modified and unmodified universal adhesive demonstrated the penetration of resin tags except for the immediate TCS group. It has been concluded that incorporation of TCS into universal adhesive seems to improve the bond strength to dentin. Six months aging has no adverse effect on bond strength of TCS incorporated universal adhesive to dentin.

## Introduction

Nowadays, preservation of precious tooth substrate becomes the keystone of modern Restorative dentistry^1^ Moreover, conservation of tooth structure plays an important role in maintaining an equilibrium between biological, mechanical, functional, and esthetical parameters. Thus, unduly cutting of tooth structure may adversely affect the pulpal tissue leading to permanent pulp damage^2^.

Currently, clinicians prefer using adhesive restorations due to their advantages in conserving tooth structure, as well as their superior esthetic and functional properties. Adhesion of resin-based restorations relay on creation of a collagen fibrils reinforced resin matrix which is referred to as hybrid layer. Therefore, obtaining a stable ‘durable’ bonding with dentin is considered as the main challenge in adhesive dentistry^3,4^.

Self-etch adhesives systems are commonly used in daily clinical practice. They incorporate bifunctional acidic primers which simultaneously condition dentin, eliminating rinsing phase. Consequently, this might lead to shortening clinical application time and significant reduction of procedural mishaps^5^.

Concurrent minimally invasive strategies promote preservation of affected ‘demineralized’ dentin layer beneath to restorations^6^. This layer is capable for remineralization over time by interaction with bioactive materials; such as glass ionomers and modified-bioactive-adhesives^7–9^. It has been reported that application of bioactive adhesive significantly enhances remineralization of repairable dentin^10^.

Bioactive materials are massively used in restorative and endodontic fields to improve healing of reversable damages, as well as maintaining pulp vitality. Tricalcium and dicalcium silicates are typical example of bioactive products utilized in our daily clinical practice. These ceramic powders are similar to Portland cement; however, they are slightly modified for medical use in dentistry. Their hydraulic setting reaction can take place at room temperature. The moisture-tolerant nature of bioactive materials allows them positively to react with moist caries-affected ‘demineralized’ dentin^11^.

Hydraulic Calcium-Silicate Cements (HCSCs) are also commonly used for several endodontic field^12^. Calcium silicate hydrate is produced where the anhydrous phase solidifies due to hydration of tricalcium silicate (TCS). Throughout the hydration process, the material may still be able to penetrate the dentinal tubules, which might result in tubule blockage^13,14^.

Nevertheless, the effect of these materials has been reported to aid in remineralization of partially demineralized dentin, their effect on bonding to tooth substrate has been controversial. Previous studies evaluated the bioactivity and potential for remineralization of adhesives containing amorphous calcium phosphate (ACP) and calcium silicate (CS) nanoparticles. The findings demonstrated that Cs and ACP nanoparticles stimulated dentin mineralization, and that including them in dentin bonding agents may improve dentin bond bio-functionalization^15^.

While previous studies have demonstrated that incorporating calcium phosphate-based particles, such as 5 and 10% w/v of β-TCP, can enhance shear bond strength (SBS)^16^, a significant research gap remains regarding the incorporation of TCS into multi-mode universal adhesive systems. Unlike phosphate-based fillers, TCS is the primary bioactive component of silicate cement,, known for its superior alkalinizing potential and hydroxyapatite-forming ability. However, the impact of TCS on the delicate chemical balance of universal adhesives, specifically its interaction with acidic monomers (e.g.10-MDP), as well as its influence on dentin hybridization, has not been widely investigated. Furthermore, there is a lack of evidence concerning the hydro-stability of such modified interfaces. Hence, this study was designed to address this gap by evaluating a low-concentration (0.5% w/v) TCS-modified universal adhesive, focusing on its microtensile bond strength (µTBS) and micromorphological patterns both at baseline and following storage in distilled water. Consequently, the null hypotheses tested that there was no significant effect of incorporation of TCS to universal adhesive on (i) µTBS to dentin and (ii) micromorphological patterns of tooth/restoration interface.

##  Material and methods

### Materials

In this study, one type of universal adhesive resin system was used (Prime & Bond universal adhesive, Dentsply Sirona, De Trey, Konstanz, Germany) as a control adhesive system. The bioactive adhesive version was prepared by incorporation of TCS powder into the universal adhesive. Furthermore, a nanohybrid resin composite (Neo Spectra ST, Dentsply Sirona, De Trey, Konstanz, Germany) was used to cover the bonded dentin surface.

### Teeth selection

A total of 40 freshly extracted caries-free first and second human molars were used in this study. The molars were collected from patients aged 45–65 years seeking extraction treatment due to periodontal disease from the Oral Surgery Department, Faculty of Dentistry, Mansoura University. The protocol of teeth collection and storage was approved by the Mansoura University Faculty of Dentistry ethical committee under approval no. A09060922. Molars were cleaned from calculus and soft-tissue deposits using a hand scaler.

### Preparation of the experimental adhesive

TCS powder with a particle size of 1 to 10 μm was incorporated into the adhesive system, it was weighed to obtain a suspension with a final concentration of 0.5% w/v^17^. The incorporation achieved by adding it to the adhesive system and a magnetic stirrer (Hot plate and magnetic stirrer; Misung Scientific Co., Korea) was employed at 200 rpm at room temperature for 30 min until a macroscopically homogeneous suspension was achieved while avoiding excessive heat generation, which could trigger premature thermal polymerization of the resin monomers. At 0.5% w/v, the low filler loading was intended to maintain the original handling characteristics and translucency for photo-polymerization.

### Sample size justification

Sample size calculation was based on mean bond strengths between different groups of treatment of the dentin surface retrieved from previous study^18^ using G power program version 3.1.9.7 to calculate sample size based on effect size of 0.35, using 2-tailed test, α error = 0.05 and power = 90.0%, the total calculated sample size will be 7 molars per each subgroup. The experimental unit of the current study was the tooth; this means that the recorded value of each group represented the mean microtensile bond strength value of the seven teeth.

### Preparation of specimens

The molars’ roots were embedded below CEJ in an acrylic resin (Acrostone, Cairo, Egypt) within cylindrical Teflon molds. The occlusal enamel and superficial dentin were removed to expose the mid-coronal dentin producing flat dentin surface. The cutting procedure was performed using a low-speed automated diamond saw (IsoMet 4000, Buehler Ltd., Lake bluff, LL, USA) under running water. The exposed dentin surfaces were subjected to 600-grit silicon carbide paper to create a standardized smear layer^19^.

### Microtensile bond strength test

Twenty-eight molars were randomly assigned into 2 groups (*n* = 14) according to the incorporation of TCS to the universal adhesive used; Group1: Control group without incorporation of TCS. Group 2: With the incorporation of TCS to the universal adhesive. Each group were subdivided into 2 subgroups (*n* = 7) according to the testing time; Sub-group1: specimens were tested immediately; Sub-group 2: specimens were stored in distilled water for 6 months till µTBS testing.

Adhesives were applied according to manufacturer’s instructions. They were applied to the dentin surface in SE mode. The adhesive was actively applied using a microbrush with continuous agitation for 20 s. Subsequently, the adhesive layer was gently air-dried using moderate air pressure for 5 s to allow solvent evaporation. The adhesive was light-cured for 20 s using a light curing unit (LED Bluephase C5, Ivoclar, Vivadent, and Amherst, NY, USA) with wavelength between 350 and 520 nm. The light intensity was monitored using a radiometer (Optilux, Kerr, USA) and measured at 450 mW/cm². After curing of the adhesive layer, a 4 mm-height composite resin block (Neo Spectra ST– Dentsply De Trey, Konstanz, Germany) was built up following the manufacturer instructions using a split Teflon mold with a central space and dimensions of (6 mm width, 6 mm length, and 4 mm height) over the adhesive-bonded flat dentin surface; each increment was compressed using a plastic condenser and light cured for 20 s using visible light curing unit with an output density of 450 mW/cm2.

After complete curing of the composite, the teflon mold was removed and then the teeth were kept in distilled water for 24 h at room temperature until the time of testing for the immediate subgroups after 24 h, and delayed subgroups were stored in distilled water in an incubator for 6 months at 37 °C.

Each specimen was longitudinally sectioned in both “x” and “y” directions across the bonded interface with the Isomet cutting machine (Isomet 4000, Buehler, Lake Bluff, IL, USA) to obtain bonded beams per tooth, each with a cross-sectional area of approximately 1 × 1 mm^2^^18^. The experimental unit of the current study was the tooth; this means that the recorded value of each group represented the average microtensile bond strength value of the seven teeth.

These beams were mounted onto a universal testing machine (Instron 3345, MA, USA) equipped with a 500 N load cell using Geraldeli’s jig. A crosshead speed of 0.5 mm/min was employed on a universal testing machine (Isomet 4000, Buehler Ltd., Lake Bluff, IL, USA) to apply tensile load until the bond between the specimen and the jig failed. The µTBS was then calculated in Megapascals (Bluehill Universal software, v4.47, Instron, MA, USA https://www.instron.com/en/products/materials-testing-software/bluehill-universal/)^18,20^.

After debonding, any fragments remaining on the Geraldeli’s jig were carefully removed using a scalpel and stored in labeled plastic cones corresponding to each specimen for further examination of the failure mode. To determine the mode of failure, the fractured sites were observed using a stereo-microscope (Olympus model SZ-PT, Tokyo, Japan) at 15x magnification. Failures were classified into three categories: (1) Adhesive failure, occurring at the dentin-adhesive interface; (2) Cohesive failure, occurring within the dentin or the resin composite; and (3) Mixed failure, involving both the interface and the surrounding substrates.

### Preparation of the specimens for micromorphological observation

Three extra molars from each subgroup were prepared with a total no. of 12 molars from all groups were obtained (*n*= 12). The samples are fixed and restored in the same manner for µTBS specimen preparation. The restored teeth were then sectioned into two semi-equal halves using a water-cooled diamond disc at low speed along the long axis of the teeth in a direction perpendicular to the resin-dentin interface (IsoMet 4000, USA)^21^. Each half was polished with silicon carbide paper with grits of 600, 1000, 1200, and 2000. The final polishing step involved the use of increasingly fine diamond pastes (6 μm, 4 μm, and 1 μm) (Metadi, Buehler, Lake Bluff, IL, USA) applied onto a polishing cloth. Any debris present on the slabs was removed by placing them in an ultrasonic bath (XH-E412 ultrasonic cleaner, Xinghua Ltd, China) for a duration of 10 min. The specimens were then stored in a saline solution at room temperature prior to undergoing an acid-base challenge. This involved exposing the specimens to a 10% ortho-phosphoric acid solution for 5 s, followed by a 5% sodium hypochlorite solution for 5 min. This acid-base treatment demineralized any dentin that had not been infiltrated with resin, allowing for dehydration of the dentin. Specimens were gold-sputter coated (SPI Module - Sputter Carbon/Gold Coater, EDEN instruments, Japan) and observed in secondary electron detection mode under an SEM (JSM6510LV, JEOL, Japan) at magnification (X500, X1000, and X2000)^22^.

### Statistical analysis

The SPSS program (IBM SPSS Statistics, IBM, Armonk, NY, USA) was used to calculate descriptive statistics. Statistical analysis of the data was done, testing the normality using the Kolmogorov–Smirnov test and the homogeneity of variances using Levene’s test. After confirming that the data met the parametric assumptions, two-way analysis of variance (two-way ANOVA) and Tukey’s post hoc test were conducted. Statistical significance was predetermined at *p* < 0.05.

## Results

### Microtensile bond strength results

**Fig. 1:**
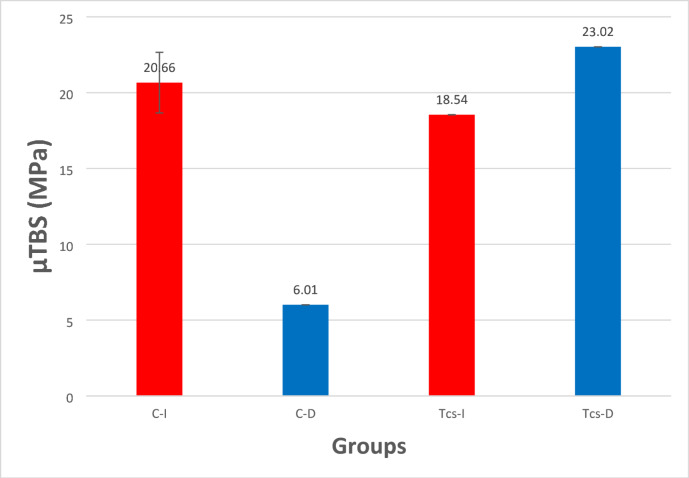
Means and standard deviations µTBS values of all groups expressed in MPa. Abbreviations; C-I: immediate control group, C-D: delayed control group, TCS-I: immediate group with TCS incorporated into the adhesive, TCS-D: delayed group with TCS incorporated into the adhesive.

The homogeneity of variances was confirmed using Levene’s test (*p* = 0.478), indicating that the data met the requirements for parametric analysis. The tooth was used as the experimental unit by averaging the µTBS values of its respective beams. The two-way ANOVA test revealed that both the adhesive type (*p* < 0.001) and time (*p* = 0.001) had a statistically significant effect on the µTBS. Moreover, the test revealed a statistically significant effect regarding the interaction of adhesive type and time on the µTBS (*p* < 0.001). The mean ± standard deviation (SD) of the µTBS in the four different subgroups are shown in Table 1, a graphical presentation of this result is presented in (Fig. 1). Comparison of µTBS data between the four subgroups, the results of µTBS, the TCS delayed subgroup showed the greatest µTBS value (23.02 ± 4.47 MPa), while the control delayed subgroup revealed the lowest µTBS value (6.01 ± 3.38 MPa). The post-hoc Tukey test was used for pairwise comparison in µTBS between the subgroups. The test showed that there was no statistically significant difference in the µTBS between the two TCS subgroups (immediate and delay) (*p* > 0.05), while there was a statistically significant difference in the two control subgroups (immediate and delay) (*p* < 0.05), while there was no statistically significant difference in the µTBS between the control immediate subgroup and TCS immediate sub-group (*p* > 0.05). However, there was a statistically significant difference in the µTBS between the two delay subgroups (control and TCS) (*p* < 0.05).

**Table 1:** µTBS values (MPa) of the tested groups.

Adhesive material	Storage	*N*	µTBS(MPa) (Mean ± SD)
Control	Immediate	7	20.66 ± 2.44^a^
	Delayed	7	6.01 ± 3.38^b^
TCS	Immediate	7	18.54 ± 3.33^a^
	Delayed	7	23.02 ± 4.47^a^

• Abbreviations; C: control group without incorporation of TCS into the Adhesive, TCS: group with TCS incorporated into the adhesive.

• Tooth is the experimental unit of the current study.

• Means followed by different letters represent significant differences according to Tukey’s test.

### Analysis of failures mode

**Fig. 2:**
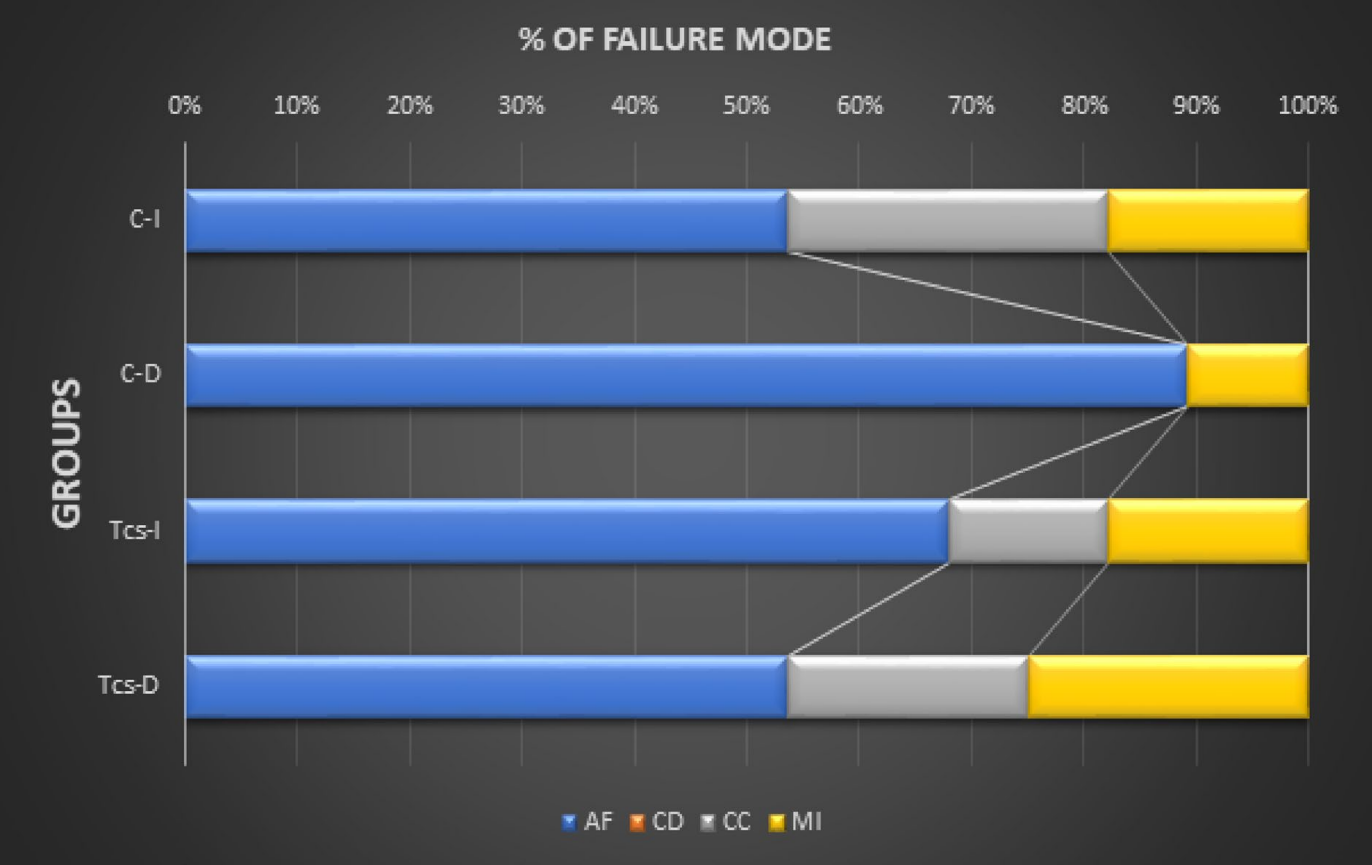
Percentage of failure pattern among the study groups.AF: adhesive failure; CD: cohesive failure in dentin; CC: cohesive failure in composite; MI: mixed failure; C-I: immediate control group, C-D: delayed control group, TCS-I: immediate group with TCS incorporated into the adhesive, TCS -D: delayed group with TCS incorporated into the adhesive.

As shown in Table 2; Fig. 2 in all groups, the predominant failure mode was adhesive failure. However, the cohesive failure mode percentage was the lowest when compared to other failure modes. Furthermore, there is a decrease in the percentage of adhesive failure in the immediate control group and delayed TCS group compared to other groups. The adhesive failure mode was commonly observed in the delayed control group while cohesive failure mode was commonly observed in the immediate control group.

**Table 2:** Number of specimens (%) according to fracture Mode of all experimental groups.

Adhesive material	Storage	Fracture pattern			
		A	CD	CC	MI
Control	Immediate	15(53.6%)	-	8(28.5%)	5(17.9%)
	Delayed	25(89.3%)	-	-	3(10.7%)
TCS	Immediate	19(67.9%)	-	4(14.2%)	5(17.9%)
	Delayed	15(53.6%)	-	6(21.4%)	7(25%)

A: adhesive fracture mode, CC: cohesive fracture mode in composite, CD: cohesive fracture mode in dentin, MI: adhesive/mixed fracture mode.

### Micromorphological evaluation

**Fig. 3:**
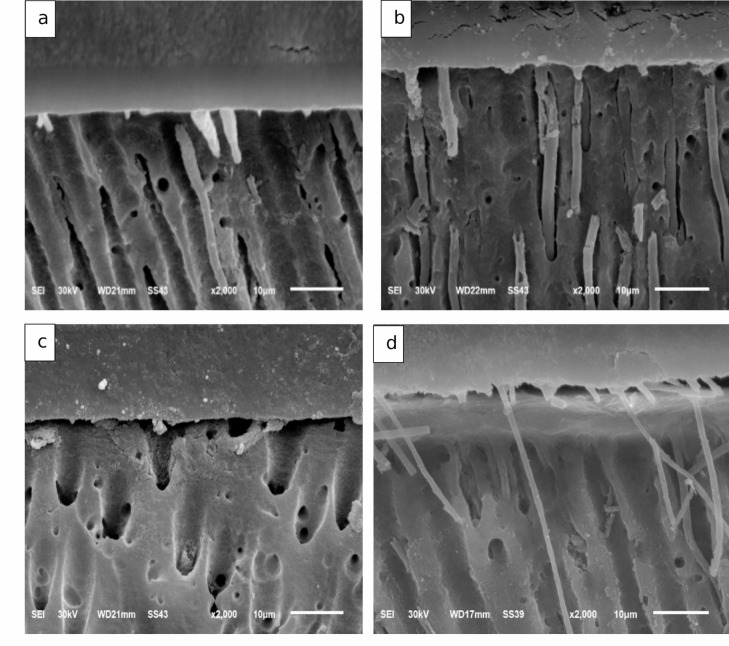
SEM micrographs showing the resin-dentin interface of all groups. (**a**) Immediate control group. (**b**) Delayed control group. (**c**) Immediate TCS group. (**d**) Delayed TCS group

All groups exhibit poor tubular penetration with short and discrete resin tags may be formed. Additionally, a very thin hybrid layer formation, or probably more correctly a poorly resin-infiltrated smear layer that significantly varies in thickness along the resin-dentin interface were observed. Resin tag penetration was observed in both modified and unmodified universal adhesive groups; however, resin tags were not clearly evident in the immediate TCS group. Control groups showed no signs of separation throughout the interface. Conversely, TCS groups showed formation of interfacial gaps at the dentin–resin interface (Fig. 3).

## Discussion

Recently, calcium silicate-based materials have received widespread acceptance in medicine and dentistry due to their biocompatibility, bioactivity and their unique ability for remineralization. Its hydrophilic property is also a significant benefit, as it may react with water at ambient temperature to produce a hydraulic setting reaction that solidifies the compound into a solid mass.

In the present study, incorporation of TCS into the universal adhesive employed through prolonged magnetic stirring and visual inspection to ensure no visible precipitates remained. A low concentration (0.5% w/v)^17,23^ used to minimize the impact on the refractive index and viscosity of the resin, aiming to preserve the original handling properties. A high filler ratio makes the adhesive resin more viscous, which reduces the amount of resin that penetrates the dentin tubules^24^. It has been observed that adhesive resin bond strengths are considerably decreased when the filler ratio exceeds 10%^25,26^.

The µTBS results of current study showed that the modified adhesive’s value increased when compared to the non-modified adhesive tested, but there was no significant difference between them immediately. However, after six months of storage, there was a significant decrease in µTBS of resin composite to dentin in the control delayed subgroup compared to the TCS delayed subgroup.

This enhancement in µTBS may be explained by the fact that resin bonding systems including particularly ca-silicate microfillers may encourage a therapeutic mineral deposition inside the hybrid layer and increase the endurance of the resin–dentin bonding^27^. It was found in a study by Profeta et al^27^. that the bonding strength of adhesives was enhanced using specially designed Portland cement microfillers. The small amount of fillers that were added and which had no impact on the altered adhesive flow or viscosity serve as evidence supporting this assumption^28^. Moreover, the period of storage in distilled water had no effect on the bonding strength. The results of the adhesive mode failure, which indicate that adhesive mode of failure happened more frequently in the non-modified adhesive group when compared to the modified adhesive group, further confirm these findings.

Furthermore, the results aligned with the work of Abuna et al^29^. who reported stable µTBS was promoted over a 6-month period by applying the Ca/P-doped adhesive with or without dentin pre-treatments using primer containing both biomimetic analogs (PAA and TMP). On the other hand, after six months, the µTBS of the filler-free adhesive and control primer dramatically dropped.

The output of Garcia et al. study^30^ were also in consistent with these findings. They concluded that the addition of α-TCP nanofiller to adhesive resins can strengthen bonds and could be a viable approach to accomplish therapeutic remineralization at the composite-dentin interface. Similar findings were reached by AlRefeai et al^31^.

A higher percentage of adhesive failures was seen in the µTBS fracture beams, which might be attributed to improved stress distribution at the small specimens’ bond interface^32^. There has been a decrease in the percentage of adhesive fractures in TCS groups for both the immediate and delayed groups. Consequently, the bond strength values in the TCS groups were supported by the mode of failure, with particular emphasis on the TCS group, where it is possible that the TCS interacted with the tooth substrate’s hydroxyapatite to increase the bond strength. This was comparable to what Molla et al^33^.. and Hamama et al^19^. concluded. They found that adhesive failure is the least frequent mode of bond failure at high bond strengths. Furthermore, a greater percentage of adhesive failures in the control delayed group were noted. In comparison to the other groups, this was linked to the lowest µTBS values (statistically significant).

In the present study, SEM evaluation provided qualitative morphological observations of the resin–dentin interface. No evident qualitative alterations in the general interfacial morphology were observed following the incorporation of a small amount of TCS. However, hybrid layer thickness and resin monomer penetration into dentinal tubules were not quantitatively measured and therefore no definitive conclusions can be drawn regarding these parameters. The absence of clearly visible resin tags in the immediate TCS group may be attributed to localized interfacial variability, differences in sectioning orientation, or early-stage interfacial adaptation.

Based on SEM observations in the current study, TCS incorporated adhesive validated appropriate dentin interaction. These resin tags were comparable to the control group. It should be mentioned that several previous studies informed that the penetration depth of resin tags in the dentinal tubules does not significantly impact the strength of the bond or the integrity of the adhesive^34–36^, even if the measurements to determine this depth were not carried out in this study. This fact might explain the reason why the bond strength was not affected despite the lack of good penetration of the resin tags into the dentinal tubules in immediate TCS group.

Moreover, de Oliveira et al.^37^, conducted a study in 2002 that could explain the absence of relationship between the adhesive system’s bonding strength and the length of the resin tags. They claimed that applying a self-etching adhesive to dentin promotes less molecular weight molecules, such as hydrophilic monomers (HEMA), to migrate deeper. Consequently, smaller molecular weight monomers that are poorly cured constitute the majority of tags, which reduces their contribution to the bond strength.

The outcomes of the current study revealed that the incorporation of TCS to the universal adhesive did not result in significantly different µTBS to dentin immediately, however, there was an increase in bond strength value in modified adhesive compared with non-modified adhesive, while it resulted in significantly different µTBS to dentin after six months of distilled water storage. The stability of the µTBS values in the TCS-modified group after water storage may be attributed to the unique hydration kinetics of silicate particles. Unlike inert fillers, TCS reacts with residual moisture within the hybrid layer to produce calcium hydroxide (Ca(OH) _2_). This byproduct not only promotes a high local pH, which has been shown to cross-link collagen and inhibit endogenous MMPs, but also provides a source of calcium ions for the potential remineralization of ‘naked’ collagen fibers that were not fully infiltrated by the resin monomers^38,39^.

SEM observations were limited to qualitative morphological assessment and were not intended to quantitatively validate the µTBS findings. Thus, the first null hypothesis that the incorporation of TCS into the universal adhesive would have no significant effect on the µTBS to dentin was rejected. Similarly, the second null hypothesis that the TCS incorporation would not influence the micromorphological patterns of the resin/dentin interface was also rejected.

Despite the improvement in bond strength observed in this study, certain limitations regarding the physicochemical characterization of the modified adhesive must be acknowledged. The incorporation of tricalcium silicate (TCS) particles into a polymer matrix can influence rheological properties, such as viscosity, and may affect polymerization kinetics due to light scattering by the inorganic filler. While the current study utilized a relatively low concentration of TCS (0.5% w/v) to mitigate these effects, the degree of conversion and the homogeneity of particle distribution were not quantitatively evaluated via FTIR or SEM/EDX. Future studies should include a comprehensive rheological and spectroscopic analysis to ensure that the addition of bioactive fillers does not compromise the adhesive’s penetration into the dentinal tubules or its long-term hydrolytic stability.

Furthermore, the aging protocol in the present study was limited to storage in distilled water. While water storage is a standardized method for evaluating hydrolytic degradation of the adhesive interface, it does not fully replicate the complex oral environment. The absence of thermocycling, mechanical loading, and pH cycling may have an impact on the results obtained. Consequently, the durability claims in this study should be interpreted as a baseline assessment of hydrolytic stability, and the clinical longevity of the bond under dynamic oral conditions requires further investigation using more complex aging models.

## Conclusions

Within the limitations and based on the outcome of present study, the µTBS test supported by analysis of failure mode and micromorphological observation of adhesive/dentin interface, the following conclusions could be drawn:

1. The incorporation of TCS into the universal adhesive seems to improve the bond strength to dentin.
2. Six months aging had no adverse effect on the bond strength of TCS incorporated universal adhesive to dentin.

## Data Availability

The datasets used and/or analyzed during the current study are available from the corresponding author on reasonable request.

## Abbreviations

ACP: Amorphous calcium phosphate
CS: Calcium silicate
hCSCs: Hydraulic calcium-silicate cements
µTBS: Microtensile bond strength
SE: Self-etch
SEM: Scanning electron microscope
SD: Standard deviation
TCS: Tricalcium silicate
